# Comparative Study of Human and Mouse Postsynaptic Proteomes Finds High Compositional Conservation and Abundance Differences for Key Synaptic Proteins

**DOI:** 10.1371/journal.pone.0046683

**Published:** 2012-10-05

**Authors:** Àlex Bayés, Mark O. Collins, Mike D. R. Croning, Louie N. van de Lagemaat, Jyoti S. Choudhary, Seth G. N. Grant

**Affiliations:** 1 Molecular Physiology of the Synapse Laboratory, Institut de Recerca de l'Hospital de la Santa Creu i Sant Pau, UAB, Barcelona, Catalonia, Spain; 2 Genes to Cognition Programme, Wellcome Trust Sanger Institute, Genome Campus, Hinxton, Cambridgeshire, United Kingdom; 3 Department of Clinical Neuroscience. School of Molecular and Clinical Medicine, University of Edinburgh, Edinburgh, United Kingdom; 4 Proteomic Mass Spectrometry, The Wellcome Trust Sanger Institute, Hinxton, Cambridgeshire, United Kingdom; University of Nebraska Medical Center, United States of America

## Abstract

Direct comparison of protein components from human and mouse excitatory synapses is important for determining the suitability of mice as models of human brain disease and to understand the evolution of the mammalian brain. The postsynaptic density is a highly complex set of proteins organized into molecular networks that play a central role in behavior and disease. We report the first direct comparison of the proteome of triplicate isolates of mouse and human cortical postsynaptic densities. The mouse postsynaptic density comprised 1556 proteins and the human one 1461. A large compositional overlap was observed; more than 70% of human postsynaptic density proteins were also observed in the mouse postsynaptic density. Quantitative analysis of postsynaptic density components in both species indicates a broadly similar profile of abundance but also shows that there is higher abundance variation between species than within species. Well known components of this synaptic structure are generally more abundant in the mouse postsynaptic density. Significant inter-species abundance differences exist in some families of key postsynaptic density proteins including glutamatergic neurotransmitter receptors and adaptor proteins. Furthermore, we have identified a closely interacting set of molecules enriched in the human postsynaptic density that could be involved in dendrite and spine structural plasticity. Understanding synapse proteome diversity within and between species will be important to further our understanding of brain complexity and disease.

## Introduction

Over the last decade the identification of synaptic proteins using mass spectrometry has transformed the view of the synapse as a relatively simple structure to one with a high degree of molecular complexity [1]. Proteomic studies from fly [2], mouse [3], [4], rat [5], [6] and human [7], [8] have identified many hundreds of postsynaptic proteins that are organized through physical interactions into multiprotein complexes and networks [9]. The overall function of these structures is to mediate the contact and communication of information between nerve cells. Furthermore, molecular signaling within the postsynaptic terminal not only plays key roles in rapid neuronal transmission of electrical activity, but also in a wide range of adaptive behaviors including learning and memory.

The first clue that the postsynaptic terminal was composed of an unusually large, or complex set of proteins came from the intense staining it produced when observed under the electron microscope [10]. Beneath the postsynaptic membrane was a dense band of material that was referred to thereafter as the postsynaptic density (PSD). Confirmation that the PSD was composed of many unknown proteins was obtained by gel electrophoresis of PSD proteins isolated by fractionation of brain homogenates [11], [12]. Our recent characterization of the PSD from the neocortex of humans (hPSD) [7] revealed the role of PSD genes in known Mendelian disorders (PSDopathies). By overlaying human genetic data onto the PSD, we found that mutations in 207 PSD genes (15% of PSD genes) cause diseases including 133 brain diseases.

It is important to compare mouse and human synapse proteomes for several reasons. The extent that mouse genetic models recapitulate features of human disease will likely be influenced by the similarity of the protein networks in which that gene functions. Synapse proteins control behavior and thus differences may provide insights into changes in species behavior. Toward validating animal models for human disease at the molecular level and towards better understanding the evolution of the mammalian synapse and brain, we report what, to our knowledge, is the first comparative proteomic study of the human and mouse PSD. Although proteomic profiling of the mouse PSD has been previously reported [4], [13], here we compare and contrast human and mouse PSDs isolated and analyzed in parallel and by identical methods. Thus differences in fractionation methods, instrument sensitivity and other parameters that differ between laboratories and inevitably introduce significant variation in the results are minimized. We compare the numbers, types and abundances of PSD proteins between these two species and present a functional classification of mammalian cortical PSD molecules. All the data generated is freely available in the G2Cdb database (http://www.genes2cognition.org/publications/human-mouse-psp).

## Materials and Methods

### Ethics Statement

Animals were treated in accordance with UK Animal Scientific Procedures Act (1986). All procedures were supervised by the Wellcome Trust Ethics Committee and the UK Home Office (Project License: 80/2337).

### Human Cortex Samples

Human cortex samples and PSD proteins used in this study had been previously described [7]. Human cortex was obtained from 9 different neurosurgical procedures. Immediately following removal from the brain, neuropathologists assessed whether it was normal or diseased tissue and only those samples that were designed as normal were used in the present study.

### Mouse Cortex Samples

In total 5 male and 5 female 6–8 week old mice, from the 129 Strain, were used in this study. Prior to isolation of postsynaptic densities cortex was dissected from the rest of the brain, including hippocampus. The 10 cortices were pooled into three groups (one group of four and two of three) to make the three replicates used in this study. All mouse and human sample processing steps were performed in parallel (at the same time) and peptide fractions from all samples analyzed back-to-back within just over a week and the performance and sensitivity was monitored throughout.

### Reagents

All chemicals were purchased from SIGMA. MOPS electrophoresis buffer and pre-casted 4–12% Bis-Tris SDS-PAGE gels were obtained from Invitrogen. Antibodies were purchased from BD bioscience (Anxa 2, 610069; Baiap2, 612674/5; Grin2B, 610416/7 and Dlg1, 610874/5), Millipore (Grin1, 05-432; Camk2a, 05-532; Grin2a 07-632; and Rac1, 05-389), Neuro Mab (Dlg2, 75-057 and Dlg3, 75-058), Affinity (Dlg4, MA1-045), Sigma-Aldrich (Anxa6, HPA009650) and AbCam (Syngap1, ab3344).

### Postsynaptic Density Fractionation

Human and mouse samples were fractionated in parallel, using the exact same methods and equipment. Postsynaptic densities were prepared as described previously [14]. Essentially, tissue was homogenized 9∶1 (v∶w) using a glass-teflon tissue grinder in a buffer containing Tris 50 mM pH 7.4, 0.3M sucrose, 5 mM EDTA, 1 mM PMSF, 2 µM Aprotinin and 2 µM Leupeptin. A first 800×g centrifugation was used to discard nuclei and cell debris, the supernatant was subjected to a second centrifugation at 16000×g to collect a pellet containing the membrane fraction. This was resuspended 5∶1(v∶w) in Tris 50 mM pH 8.1, 5 mM EDTA, 1 mM PMSF, 2 µM Aprotinin and 2 µM Leupeptin, and chilled in ice for 45 minutes. Solid sucrose was added to a final 34% (w/w) concentration. A sucrose gradient was prepared with equal volumes of the following layers (bottom to top): sample, Tris 50 mM pH 7.4-0.85M sucrose and Tris 50 mM pH 7.4-0.3M sucrose. The gradient was centrifuged for 2 hours at 60000×g. The inter-phase between 34% and 28.5% sucrose was collected, diluted to 10% sucrose with Tris 50 mM pH 7.4 and subjected to a 30 minutes centrifugation at 48000×g. Pellet was resuspended in 3 ml of Tris 50 mM pH 7.4 to generate the synaptosomal fraction. An equal volume of 3% Triton X-100 was added to the synaptosomal fraction and chilled for 30 minutes in ice. Sample was layered on top of 30 ml of Tris 50 mM pH 7.4-0.85M sucrose and centrifuged at 104000×g for 1 hour to obtain the PSD fractions as a pellet.

### LC-MS/MS Analysis on Mouse and Human PSD Fractions

All processing of human and mouse cortical PSD fractions was performed in parallel, using the same methods and equipment. Our experimental analysis of the Human PSD has been described in a previous study [7]. 25 µg of cortical PSD protein from each purification was separated by SDS-PAGE electrophoresis using a NuPAGE 4–12% Bis-Tris gel (1.5 mm×10 well, Invitrogen) and MOPS buffer. The gel was stained overnight with colloidal Coomassie blue (Sigma). Each lane was excised into 32 bands that were in-gel digested overnight using trypsin (sequencing grade; Roche). Peptides were extracted from gel bands twice with 50% acetonitrile/0.5% formic acid and dried in a SpeedVac (Thermo). Peptides were resuspended using 0.5% formic acid were analyzed online using an Ultimate 3000 Nano/Capillary LC System (Dionex) coupled to an LTQ FT Ultra hybrid mass spectrometer (Thermo Electron) equipped with a nanospray ion source. Peptides were desalted on-line using a micro-Precolumn cartridge (C18 Pepmap 100, LC Packings) and then separated using a 45 min RP gradient (4–32% acetonitrile/0.1% formic acid) on a BEH C18 analytical column (2.1 mm×100 mm, 1.7 µm) (Waters).

The mass spectrometer was operated in standard data dependent acquisition mode controlled by Xcalibur 2.0. The instrument was operated with a cycle of one MS (in the FTICR cell) acquired at a resolution of 100,000 at m/z 400, with the top five most abundant multiply-charged ions in a given chromatographic window subjected to MS/MS fragmentation in the linear ion trap. A total of 96 LC-MS/MS analyses were performed and 108,608 MS/MS spectra were acquired. All data were processed using BioWorks V3.3 (Thermo Electron) and searched using Mascot server 2.2 (Matrix Science) against a Human IPI sequence database (June, 2007) using following search parameters: trypsin with a maximum of 2 mis-cleavages, 20 ppm for MS mass tolerance, 0.5 Da for MS/MS mass tolerance, with 3 variable modifications of Acetyl (Protein N-term), Carbamidomethyl (C), and Oxidation (M). False discovery rates determined by reverse database searches and empirical analyses of the distributions of mass deviation and Mascot Ion Scores were used to establish score and mass accuracy filters. Application of these filters to this dataset resulted in a <1% false discovery rate as assessed by reverse database searching. Protein hits from all datasets were BLAST-clustered using a threshold of 95% sequence identity over at least 50% of sequence length.

Our criteria for inclusion of a protein in the mouse PSD was the same as that used for human [7]: a protein had to be identified with at least two peptides in one of the replicates or with 1 peptide in all of them and consensus mPSD proteins were identified with a minimum of 2 peptides in triplicate.

### Protein Quantification Using Intensity Based Absolute Quantification (IBAQ)

iBAQ values were calculated using MaxQuant 1.2.0.18 [15] as described [16].

In order to perform cross-species label-free quantification, protein intensities and iBAQ values were calculated using only peptides that were shared between species, that is, peptides with 100% sequence conservation. This was achieved by performing protein identification and protein intensity calculations for human PSD data (in MaxQuant) using a mouse protein sequence database, and for mouse PSD data using a human protein sequence database. Only proteins for which a quantitative value could be measured for all replicates in both species were considered for the analysis. Protein iBAQ values for each individual PSD dataset were normalized and log2 transformed. Protein expression differences between human and mouse PSD datasets were identified using t-testing with a Permutation-based FDR (0.01) to correct for multiple hypothesis testing (Perseus v1.2.0.17). Hierarchal clustering analysis was performed using Perseus v1.2.0.17 using Euclidean distance.

### Proteomic Data Integration

In order to compare the sets of PSD proteins identified from mouse and human cortical samples, we mapped the IPI protein identifiers back to their respective genomes via UniProt, or sequence search, using the Ensembl gene annotation database [17]. In the cases where a clear 1∶1 orthologue was not predicted by Ensembl, we manually searched by gene symbol on the Mouse Genome Informatics (MGI [18]) and HUGO Gene Nomenclature Committee (HGNC [19]) database websites in an attempt to find the orthologous pair.

### Analysis of Sequence Conservation in Coding Sequences of PSD Genes

dN and dS values were obtained from Ensembl version 62 [20] and dN/dS ratios computed (Table S10). Differences between dN/dS distributions between dataset were computed using the Mann-Whitney U test.

### Bioinformatic Functional Analysis of PSD Proteins

All functional analyses were done using the Panther 7.0 Classification System [21] and its expression analysis tool [22]. Biological Process terms were used as defined by the Gene Ontology Consortium [23]. The Panther terms ‘Protein Class’ and ‘Pathway’ were also used. In all cases binomial statistics were used to find enriched terms only amongst consensus PSD proteins. To correct for multiple testing the Benjamini-Hochberg false discovery rate procedure was applied. All enrichment analysis were done using the human genome as a background set.

### Interaction Network

Protein interactions were obtained from the database of protein associations STRING [24]. Interactions with a confidence score below 0.4 were not considered for the final map and proteins without interactions were excluded. The interaction map was built with BioLayout Express [25]. Edge color reflects confidence score; red being maximum confidence (1) and blue minimum (0.4). Node clustering was done with BioLayout using the Markov Clustering Algorithm.

## Results

### Overall Similarity of the Mouse and Human Postsynaptic Proteomes

**Figure 1:**
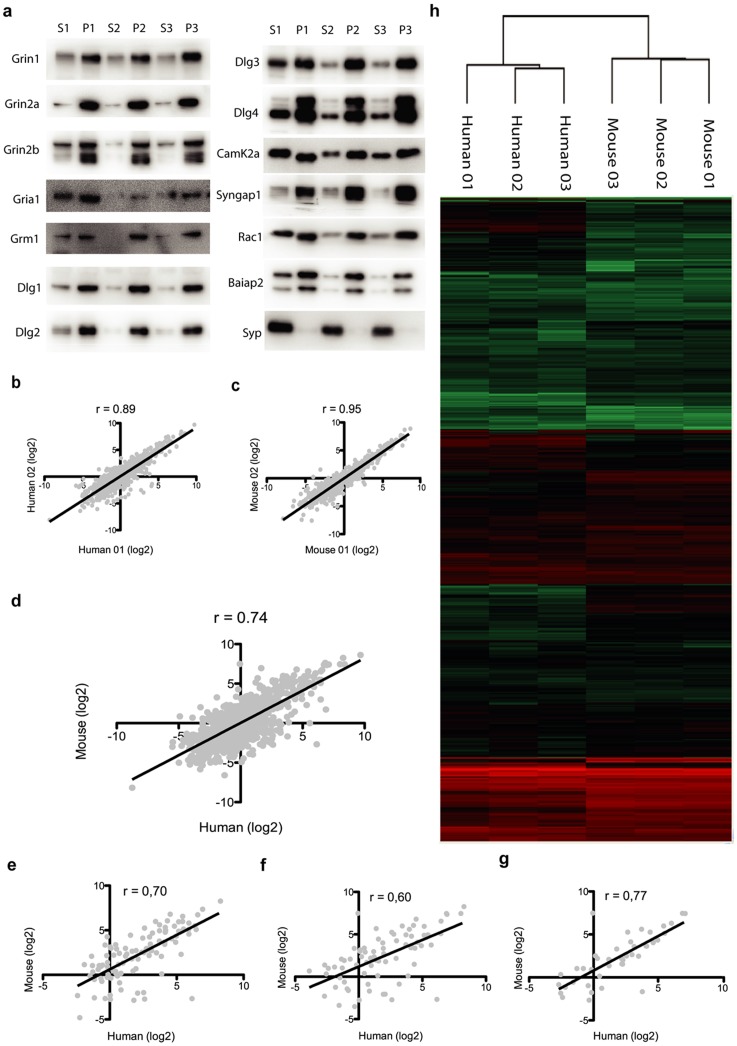
Validation of fractionation method and quantitative comparison of mouse and human cortical PSDs. **a.** Immunoblots of known postsynaptic density components. Mouse synaptosomes (S) and PSD (P) fractions are analyzed for each of the three replicates. All proteins show enrichment in the PSD compared to synaptosomes. The presynaptic marker synaptophysin (Syn) is only detected in the synaptosomal fraction. Only genes identified in triplicate in both species were considered for the quantitative analysis. Correlations are calculated based on linear models. Coefficients of correlations are represented by r. Examples of intra-species protein abundance correlation: **b.** human PSD first replica against second; **c.** mouse PSD first replica against second. **d.** Plot of average values of PSD proteins abundance from human and mouse cortex. **e.** Protein abundance correlation of PSD components found in the NMDA receptor complex [3]; the PSD-95 complex [26] (**f**), and the mGluR5 complex [27] (**g**). **h.** Clustering of PSD protein abundance values for human and mouse replicates.

Triplicate postsynaptic density (PSD) fractions were isolated from whole mouse cortex (mPSD) and human cortex (hPSD; reported earlier, pooled from 9 different regions from the temporal, frontal and parietal lobes [7]). Here we report the data from the mPSD and its comparison with the human. In all triplicate mPSD samples immunoblotting of synaptosomal and PSD fractions for well-known PSD components (Figure 1a) revealed clear enrichment for all tested PSD proteins; conversely, the pre-synaptic marker synaptophysin was depleted from PSD fractions.

Subsequent proteomic profiling of the three mPSD samples by high resolution tandem mass spectrometry (MS) identified a total of 1556 proteins of which 984 were found in triplicate and constitute the consensus mPSD (Table S1). These numbers are similar to what was found in the hPSD, which showed 1461 total and 748 consensus proteins. Using orthologous relationships between human and mouse proteins, we found ∼70% overlap between human and mouse PSD lists, leading to a combined count of 1998 cortical PSD proteins identified in humans and mouse.

To quantify intra- and interspecies PSD protein abundance differences we performed ‘intensity-based absolute quantification’ (iBAQ) using raw MS data to measure protein abundance [16]. Briefly, it allows the relative abundance of proteins to be calculated by summing all peptide 3D peak intensities detected for a given protein in a set of LC-MS/MS experiments and normalizing it by the number of theoretically observable peptides under the experimental conditions used. Only proteins for which a quantitative value could be measured for all replicates in both species were considered for the analysis (see methods, Table S2) and iBAQ values for each replicate were normalized to account for any discrepancies in total protein amounts loaded on gels.

High correlations were observed between intra-species protein abundances (human, r = 0.89±0.006 and mouse r = 0.95±0.01; Figure 1b,c), clearly indicating the reproducibility and validity of the protein quantification approach used. This is important for the human set as it was derived from nine samples from different cortical locations, and confirms that the mixing of these samples resulted in three equivalent/averaged cortical PSD samples. Between species, the correlation of overall abundance was also significant (r = 0.74, p<0.0001 Fig. 1d) although substantially lower: in other words, inter-species differences in abundance are much larger than intra-species differences.

Glutamate receptors and scaffold proteins are constituents of well-known protein complexes embedded in the PSD. It is of interest to ask if the proteins that form these complexes show a different degree of variation between humans and mice than the rest of the PSD. To address this question, we computed the correlation coefficients between human and mouse for subsets of PSD proteins belonging to the NMDA receptor [3], PSD-95 [26] and mGluR5 [27] complexes (Fig.1e–g and Table S2). This analysis showed that the abundance of components of these complexes were not significantly different (unpaired t-test with Welch's correction, p>0.05) from the whole PSD datasets. Hierarchical clustering analysis of all replicates also indicates that mouse and human samples group together (Fig. 1h), bolstering the observation of higher similarity of intra-specific protein abundance.

**Figure 2:**
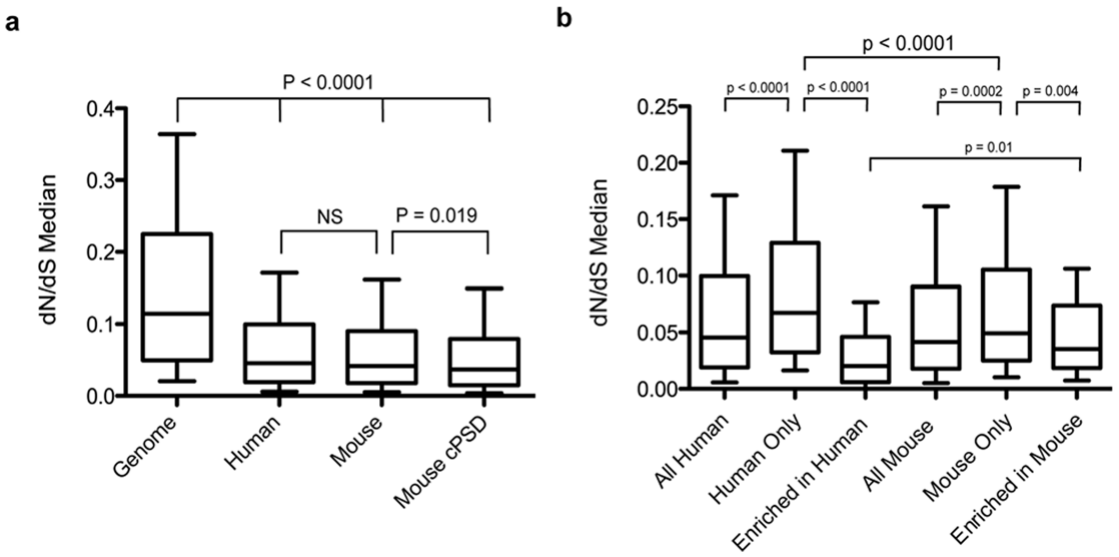
Analysis of evolutionary conservation of PSD proteins in mouse and humans. **a.** Median dN/dS of proteins identified in the human PSD, mouse PSD and mouse consensus set (cPSD) compared with the median genomic value. **b.** Comparison of dN/dS values in proteins exclusive or enriched in one species with all PSD proteins.

Based on hPSD data we had previously shown that PSD protein coding sequence has been under very strong evolutionary (purifying) constraint since mouse and human diverged 90 million years ago [7]. To re-evaluate this observation with the mouse proteomic data, which has over 500 proteins not detected in humans (Figure 2a), we again used the dN/dS ratio to measure protein conservation. While dN measures the rate of mutations causing amino acid substitutions, dS accounts for synonymous changes and represents the background rate of DNA change. Low dN/dS values indicate evolutionary conservation while high values imply the opposite [28]. The mPSD dN/dS ratio was significantly lower than that of the whole genome while equivalent to the hPSD (Figure 2a). Furthermore, the consensus set of mouse postsynaptic molecules was more conserved than the whole set, as we previously found in humans [7]. These data confirms that natural selection exerts strong protein sequence constraint in both mouse and human PSD proteins. We also found that proteins exclusive to each species have a significantly higher dN/dS than the rest of PSD genes (human p<0.0001; mouse p = 0.0002) or genes differentially expressed between species (human p<0.0001; mouse p = 0.004; Figure 2b).

### Species Expression Differences in Key PSD Proteins

It is important to ask if differences in the composition of the hPSD and mPSD arise as a result of differences in expression of particular proteins. Addressing this issue is challenging given that the human cortex is a thousand-fold larger than the mouse cortex and our limited sampling of 9 human regions is not expected to capture the full range of diversity within the cortex. Nevertheless, consistent with the observed similarity of the triplicate hPSD samples, a recent systematic study of transcriptome expression in human brain regions (including many areas of the cortex) show that different cortical regions have generally very similar overall gene expression patterns [29]. For the purposes of the next analyses we will make the assumption that our 9 human samples are representative of all human cortical regions and examine the null hypothesis that the hPSD and mPSD are the same.

**Figure 3:**
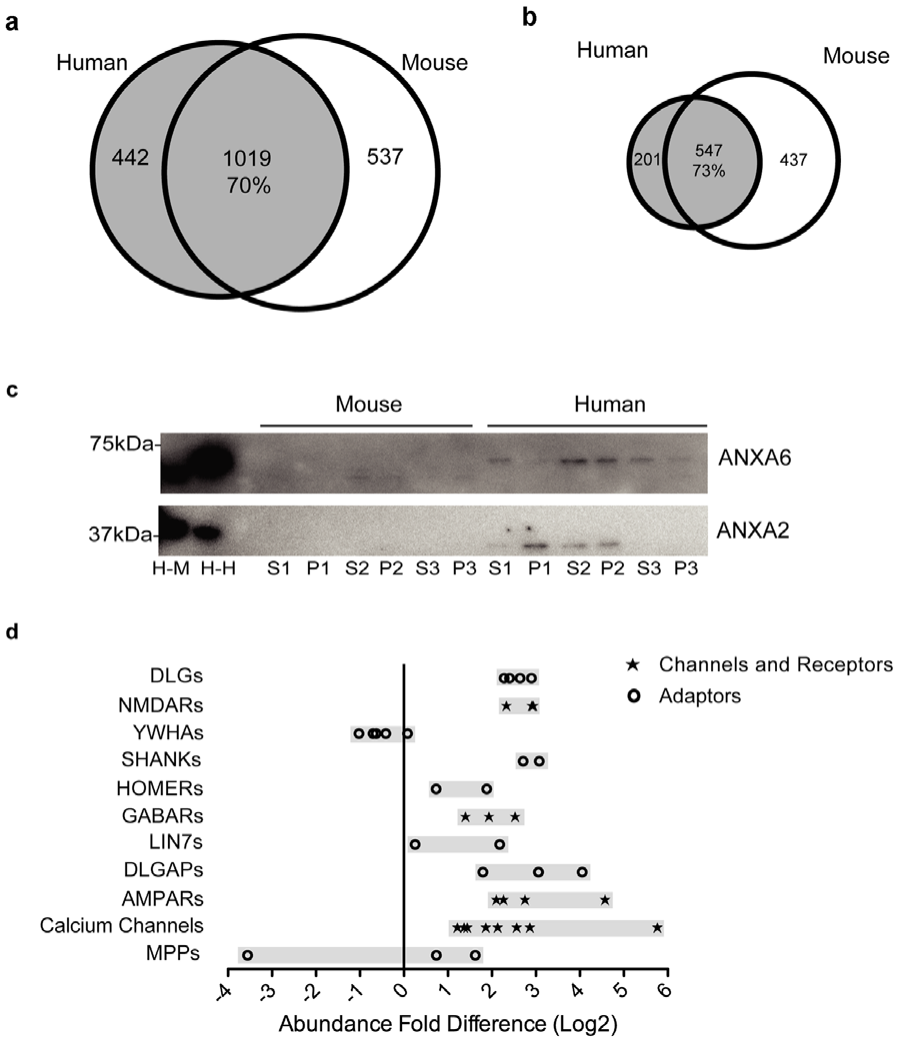
Inter-species differences on postsynaptic density proteins. **a.** Venn diagrams showing the total amount of proteins in mPSD and hPSD as well as the overlap between them relative to the hPSD. **b.** Venn diagrams showing the proteins identified in triplicate for the mPSD and hPSD, the overlap relative to hPSD between the two sets is also shown. **c.** Immunoblot validation of proteins identified only in the hPSD by mass spectrometry. The following samples were analyzed: H-M, mouse brain homogenate; H-H, human brain homogenate; and SX, each of the three synaptosomal fractions, PX, each of the three postsynaptic density fractions for both species. **d.** The abundance fold difference (AFD) between mouse and human is shown for members of adaptors, channels and neurotransmitter receptors protein families. AFD is calculated as the mouse to human ratio of normalized and log2 transformed average abundance values. For each protein family the number of proteins displayed is given in brackets.

As referred to above, comparison of the human and mouse PSD lists revealed that ∼70% of the total and 73% of the consensus hPSD proteins were present in the mouse PSD (Figure 3a,b and Table S1). We selected two of the proteins that were only identified in the hPSD (Annexin 2 and 6) by MS and validated that they were in fact specific to the human PSD by western blot (Figure 3c) thereby reinforcing observations based on MS data alone. We next asked if the ∼30% of PSD proteins found in the postsynaptic density of only one of the two species (Table S3) might confer some functional difference. To address this question, we compared their functional properties using the Panther Classification System [21]. Most protein classes showed no significant difference between species (Table S4), suggesting that the functional similarity between human and mouse PSD extends beyond the 70% of identical proteins. However, some functional groups were differentially enriched in one of the species (Table 1): for example a larger number of ion channels, particularly calcium channels, as well as molecules related to protein translation were found in the mPSD. Conversely, the hPSD showed enrichment in cytoskeletal components and some enzyme types (i. e. reductases, transferases, dehydrogenases; Table 1). Although protein kinases (PK) were enriched amongst all human PSD proteins, this was not observed if only consensus hPSD molecules were considered (Table 1).

**Table 1:** Significantly enriched protein classes of species-specific PSD proteins.

	PSD proteins		Consensus PSD proteins		Enriched in
PANTHER Protein Class	Proteins Identified	p-value	Proteins Identified	p-value	
cytoskeletal protein	36	1.3E−09	16	3.6E−07	Human
actin family cytoskeletal protein	22	7.6E−08	10	3.4E−05	Human
oxidoreductase	39	5.2E−09	13	5.1E−04	Human
Dehydrogenase	22	4.5E−08	8	8.9E−04	Human
non-motor actin binding protein	14	3.4E−06	6	1.4E−03	Human
Reductase	8	1.1E−03	4	1.4E−02	Human
Transferase	70	1.4E−08	18	2.5E−02	Human
microtubule family cytoskeletal protein	11	5.9E−03	5	3.3E−02	Human
transfer/carrier protein	14	8.5E−03	6	4.3E−02	Human
membrane trafficking regulatory protein	16	4.7E−08	4	4.3E−02	Human
nucleotide kinase	10	3.4E−06	3	4.3E−02	Human
membrane traffic protein	27	4.7E−08	5	3.0E−01	Human
Kinase	40	1.2E−07	8	3.0E−01	Human
protein kinase	25	1.2E−03	5	7.6E−01	Human
enzyme modulator	33	6.5E−03	7	7.9E−01	Human
G-protein modulator	15	8.5E−03	1	1.0E+00	Human
ribosomal protein	29	8.0E−13	16	2.3E−09	Mouse
calcium channel	7	2.9E−04	6	3.9E−05	Mouse
RNA binding protein	60	1.2E−13	20	3.9E−04	Mouse
ion channel	31	3.7E−08	12	2.1E−03	Mouse
nucleic acid binding	78	6.7E−09	28	2.5E−03	Mouse
SNARE protein	8	1.1E−04	4	9.4E−03	Mouse
Transporter	57	4.1E−06	19	8.8E−02	Mouse
ligand-gated ion channel	11	9.6E−04	4	2.2E−01	Mouse

To further pursue the possibility that protein expression levels could confer functional difference between human and mouse, we examined the abundance of proteins found in both species using iBAQ values. Of all proteins found in both species ∼8% were differentially expressed (p<0.05) in the human set and ∼10% in the mouse set (Table S2). Surprisingly, many important components of the PSD identified by previous rodent studies were amongst the proteins with greatest inter-species abundance differences, being almost in all cases more abundant in mouse (Table 2). Many proteins overrepresented or exclusive to the mouse PSD are relevant to synaptic transmission as shown by an analysis of gene ontology (Table S5). Interestingly, the abundance fold difference (AFD) between mouse and human showed considerable variation between canonical PSD molecules. For example, CAMK2A and CAMK2D have similar expression level in both species while PSD95 (DLG4) is almost 5-fold more abundant in the mouse PSD (Table 2). Moreover, the 4 members of the PSD95 family (DLG1-4) are on average 6-fold more abundant in the mouse PSD (average AFD: 5.96±1.2; Figure 3d) while another class of PSD scaffold proteins, the DLGAPs, showed more individual variability (i.e. DLGAP1 AFD = 3.4 while DLGAP4 AFD = 16.6, Figure 3d). This was also the case for subunits of the AMPA and NMDA type glutamate receptors (AMPAR and NMDAR respectively). AMPAR subunit's AFD was very variable; (average AFD: 9.9±9.3) while NMDAR subunit's abundance difference was less (average AFD: 6.7±1.5). Within the PSD are MAGUK Associated Signaling Complexes (MASC) that contains PSD-95 family, DLGAPs, NMDAR and over 100 other proteins [26] and the individual components show wide variability (average AFD: 5.8±21.5). Altogether our data suggests that there are important abundance difference in the cortex of human and mouse for some proteins key to synaptic biology.

**Table 2:** Abundance of canonical PSD components as defined by studies with rodent brains.

Appr Gene (m)	Appr Gene (h)	Abundance Fold Difference (Mouse/Human)	Significant Enrichment (p<0.05)	Appr Gene (m)	Appr Gene (h)	Abundance Fold Difference (Mouse/Human)	Significant Enrichment (p<0.05)
Ablim1	ABLIM1	1.3		Gria3	GRIA3	4.3	Mouse
Baiap2	BAIAP2	3.1		Gria4	GRIA4	23.8	Mouse
Begain	BEGAIN	10.4	Mouse	Grin1	GRIN1	7.7	Mouse
Cacng2	CACNG2	2.6		Grin2a	GRIN2A	5	Mouse
Camk2a	CAMK2A	1		Grin2b	GRIN2B	7.5	
Camk2b	CAMK2B	3.4		Homer1	HOMER1	3.7	Mouse
Camk2d	CAMK2D	0.9		Homer2	HOMER2	1.7	
Capza2	CAPZA2	1.6		Iqsec1	IQSEC1	2.4	
Cntnap1	CNTNAP1	0.5		Iqsec2	IQSEC2	2.7	Mouse
Cntnap2	CNTNAP2	2.1		Kalrn	KALRN	8.3	Mouse
Dlg1	DLG1	5.3	Mouse	Kcnab2	KCNAB2	4.4	
Dlg2	DLG2	6.3	Mouse	Kcnq2	KCNQ2	0.9	
Dlg3	DLG3	7.5	Mouse	Lgi1	LGI1	11	Mouse
Dlg4	DLG4	4.9		Magi2	MAGI2	0.5	
Dlgap1	DLGAP1	3.5	Mouse	Nlgn2	NLGN2	4.4	
Dlgap2	DLGAP2	8.3	Mouse	Nrxn1	NRXN1	1.5	
Dlgap3	DLGAP3	8.3	Mouse	Nrxn3	NRXN3	3.8	Mouse
Dlgap4	DLGAP4	16.6	Mouse	Rac1	RAC1	1.7	
Gabbr1	GABBR1	5.8		Shank2	SHANK2	8.4	Mouse
Gabbr2	GABBR2	3.8	Mouse	Shank3	SHANK3	6.5	Mouse
Gabra1	GABRA1	2.6		Syngap1	SYNGAP1	5.9	Mouse
Gnas	GNAS	1.1		Ywhae	YWHAE	1	
Gria1	GRIA1	6.7		Ywhah	YWHAH	0.5	Human
Gria2	GRIA2	4.8	Mouse	Ywhaq	YWHAQ	0.6	

Mouse to human abundance difference of proteins known to be key components of the postsynaptic density, as derived from studies on mouse and rat brains. Abundance is expressed as the fold of mouse to human average abundance values.

### Species Differences in Morphogenesis and Semaphorin Signaling

**Figure 4:**
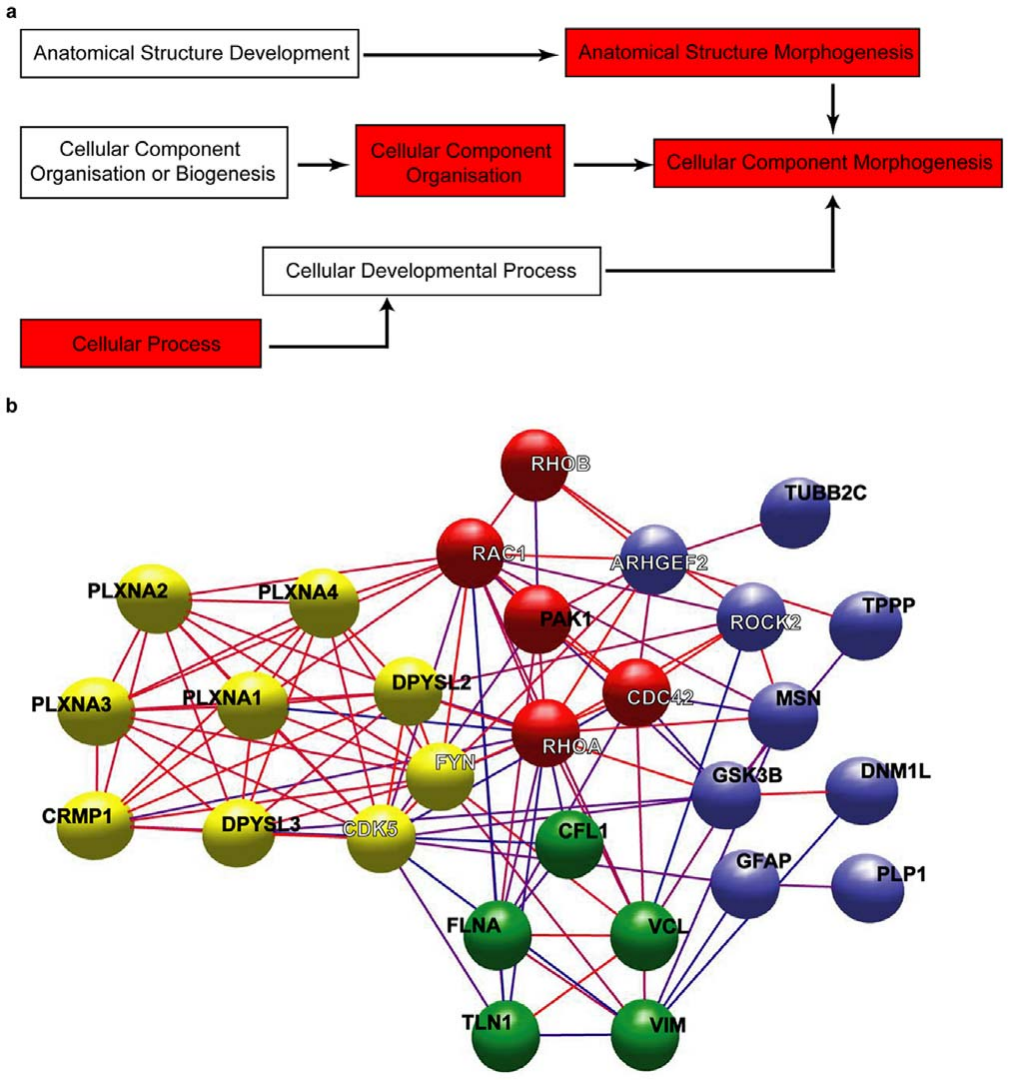
Functional analysis of proteins enriched in the human PSD. **a.** Ontology hierarchical tree leading to the Biological Process ‘Cellular Component Morphogenesis’. In red are the terms significantly enriched in proteins abundant in the human PSD. **b.** Molecular interaction map of proteins from the human PSD that belong to the Biological Process ‘Cellular Component Morphogenesis’ (CCM) or are known to be involved in semaphorin signaling. Only CCM proteins enriched in the human PSD are considered. Some proteins related to semaphorin signaling are not enriched in either species, these are identified with a white name. Proteins highly interconnected are represented in clusters of different colors. Clustering was done using the Markov Clustering Algorithm (MCL) in BioLayout Express 3D [24]. Node edges are coloured according to a confidence score, from red (100% confidence) to blue (40% confidence).

To gain further insight into functions that might distinguish human and mouse synapses, we performed gene ontology (GO) analysis. Enrichment in GO categories revealed the Biological Process category ‘cellular component morphogenesis’ amongst the most enriched categories by genes exclusive or enriched in the hPSD (p = 0.0004); other terms hierarchically related to it also appeared enriched in this analysis (Figure 4a and Table S6). Genes enriched or exclusive to the human PSD also showed enrichment in the pathway ‘axon guidance mediated by semaphorins’ (p = 0.01; Table S5).

To further explore the enrichment of semaphorin function in humans we examined the A Plexins, which bind Semaphorins 3A and 3F [30]. All four A Plexins showed greater abundance in hPSD compared to mPSD: PLXNA2 and PLXNA3 were exclusive to hPSD and PLXNA1 and PLXNA4 were over two-fold more abundant in hPSD (Table S7). Plexins interact with the family of collapsin response mediator proteins (CRMPs, currently named DPYSLs) [31]: four of the five members of this family are in the PSD, all being much more abundant in human (Table S7).

We then asked if the molecules enriched in the human PSD related to semaphorin signaling or to cellular component morphogenesis, could form a functional complex within the PSD. To address this question, we searched for protein interactions between these molecules (Table S7) and plotted them into an interaction network (Figure 4b). Most of the molecules (28/34) interact with other members of this group and overall the interaction map is highly interconnected, suggesting that this is a set of closely interacting molecules that might constitute a PSD sub-structure that is more abundant in the human cortical PSD. Further studies will be required to determine if there are biologically relevant differences in semphorin signaling in mouse and human tissue.

### Bioinformatic Functional Analysis of the Mammalian Cortical PSD

**Figure 5:**
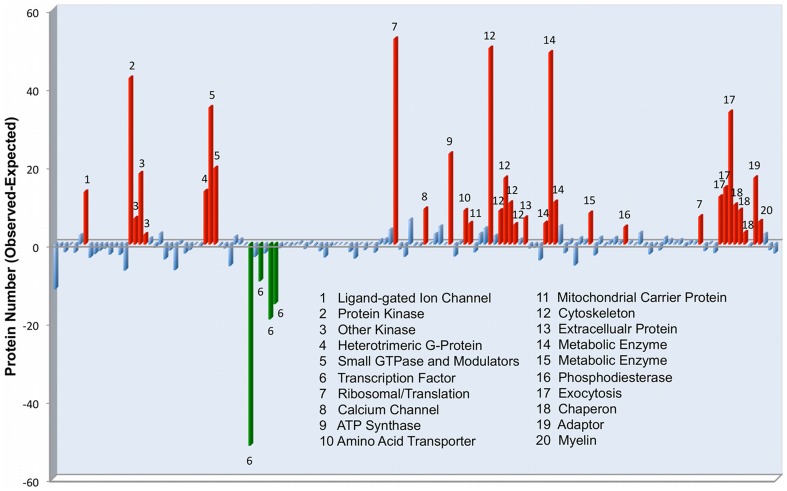
Functional protein groups with the mammalian cortical PSD. The mammalian cortical PSD was defined by combining all proteins identified in mouse and human. This set of PSD proteins were then functionally classified using the Panther Classification descriptor ‘Protein Class’. Enrichment analysis was done comparing the number of PSD proteins per class with the genome. The Panther Protein Class ontology is hierarchically organized, only end-branch classes are displayed to remove redundancy from the graph (all data in Table S8). Protein classes that did not show difference with the genome after correction for multiple testing are shown in blue. Classes overrepresented are shown in red and classes underrepresented are shown in green.

Considering together the two PSD structures derived from human and mouse cortex the total number of proteins identified is 1998 (Table S1). This is an unusually large number for a biological supramolecular protein complex, around 10% of the human or mouse genomes, and therefore it is interesting to study its main functional properties as a way to understand its overall organization. The functional analysis was performed with the Panther Classification System, which classifies proteins according to predicted function (see methods). Although most Panther Protein Classes were represented by some of the 1998 PSD proteins only ∼30% of them were significantly over or underrepresented with PSD proteins when compared with the human genome (Figure 5 and Table S8). These enrichments suggest that the mammalian PSD is a specialized molecular machine.

Signaling pathways related to the metabotropic and ionotropic glutamate receptors were amongst the most enriched, proving the validity of the methodology used. Amongst signaling molecules protein kinases, small GTPases and G-proteins are all enriched in the PSD compared to the genome; while others, such as cyclases and protein phosphatases were not (Table S8). Interestingly, although the PSD has signaling components from many different pathways, not all these pathways are equally represented in the PSD as reflected by the enrichment p-values (see methods, Table S9). Molecules involved in translation (i.e., RNA binding protein, p = 2.48×10^−10^; ribosomal protein, p = 2.8×10^−20^), amino acid transport, exocytosis and calcium channels (p = 2.8×10^−4^) are also found to be significantly enriched in the postsynaptic machinery (Figure 5). Interestingly, other cation channels, such as sodium or potassium are not enriched in the PSD; likely reflecting the more central role of calcium in the biology of the spine. Some protein classes were also underrepresented in the PSD when compared with the genome (Figure 5 and Table S8). Particularly significant is the depletion of transcription factors (p = 1.9×10^−42^); molecules related to the immune system (i.e. cytokines p = 9.2×10^−3^); extracellular signaling molecules such as peptide hormones (p = 0.018), and proteases (p = 0.025) and their inhibitors (p = 0.024).

Signaling pathway enrichment analysis (Table S9) revealed overrepresentation in the PSD. For instance, the WNT and PI3K signaling pathways, which have been proposed to be involved in the pathophysiology of schizophrenia [32], [33], [34], are enriched in the PSD, as are the cadherin, EGF and Hedgehog signaling pathways. In contrast FGF, TGFβ, p53, IGF, VEGF, PDGF or Toll-Receptor signaling pathways, amongst others, are not overrepresented in the PSD proteins, suggesting that they might have a lesser role in postsynaptic biology.

Finally we also analyzed whether molecules related to the main neurodegenerative diseases were present in the PSD. Interestingly, only some of these diseases show and overrepresentation of their related proteins in the PSD. Molecules involved in Huntington's (p = 7.3×10^−15^) and Parkinson's (p = 1.1×10^−13^) diseases are very much enriched in the PSD, while those related to Alzheimer's (‘Alzheimer disease-presenilin pathway’, p = 0.4 and ‘Alzheimer disease-amyloid secretase pathway’, p = 0.05; Table S9) were not significantly enriched within PSD genes. These results suggest that dysfunction of the PSD could be more closely linked to the pathology of Huntington's, which has already been proposed as a synaptopathy [35], or Parkinson's.

## Discussion

This study reports the first parallel comparative proteomic analysis of the human and mouse postsynaptic density, a key structure underlying brain function and animal behavior. Using samples of cortex obtained from mice and humans, the PSD was isolated and its protein composition interrogated by MS. A similar proteome complexity was observed in mouse (1556 proteins in total PSD, 984 in consensus) and human (1461 total, 748 consensus). The finding that PSD molecules have been under a very strong evolutionary purifying constraint since the divergence of primate and rodent lineages [7] is further confirmed when analyzing the molecules from mouse.

The molecular composition of the PSD in human and mouse cortex is very similar, with more than 70% of hPSD proteins in the mPSD. Computational functional analysis indicates that genes specific to each species (in the remaining 30%) do not introduce overall differential functional properties between species, suggesting that the PSD is even more similar at the functional level than at the composition level.

We have measured the protein abundance of PSD proteins identified in mouse and human cortex and its comparison indicates that they are broadly correlated, which supports the findings obtained from transcriptomic data [29], [36], [37]. Nevertheless, we have shown that protein abundance is much more variable between species than within species, suggesting that some of the mechanisms that control protein expression and turnover in cortical synapses vary between species. Since we have shown that a very similar set of proteins are present in the human and mouse PSD and that these have been under strong evolutionary constraint, the differences between species are more likely to be consequence of gene regulatory differences that influence expression and abundance.

Most well-known PSD components defined in rodent studies are more abundant in the mouse PSD. For instance, the molecules associated with PSD95 forming signaling complexes [26] have, on average, an abundance fold difference between mouse and human (AFD) of 5.8±21.5. This enrichment of canonical postsynaptic molecules in the mouse PSD could be a consequence of the mouse cortical anatomy; as mouse cortex has been reported to have a spine density up to three times higher than that of human cortex [38]. This could affect the fractionation efficiency, yielding a more enriched PSD preparation from mouse tissue. Nevertheless, if this was the sole reason behind abundance differences between species we should observe a more or less constant AFD between species, and this is not the case. The AFD between mouse and human varies considerably, even for key postsynaptic molecules, as indicated by the high standard deviation of the average AFD of PSD95 complex components. Interestingly, we have seen that components of some families of postsynaptic proteins show little AFD variation while the components of other families vary much more. These large inter-species abundance differences in individual proteins could translate into important functional differences and are the most important source of disparity we have observed between the mouse and human PSD proteomes.

Amongst proteins exclusive or enriched in the human PSD we have identified a set of molecules that are highly interconnected and are involved in cellular morphogenesis and semaphorin signaling. Semaphorins are better known for their role in axon guidance but several recent studies have elucidated their function in dendrite and spine morphology [30], [39], [40], [41], [42]. The fact that some of these molecules (DPYSL2 or PLXNA4) have also been identified in human postsynaptic complexes isolated by affinity purification (data not shown) strengthens their likelihood of being true PSD components. We propose that these molecules interact together as a protein complex within the PSD and are involved in processing semaphorin signaling at spines. Their higher abundance suggests that they might have a different or more relevant role in humans. It has been shown that the expression of semaphorin receptors in mouse cortex is very much reduced in adulthood [39], [40] while in human adult cortex expression remains high (Allen Brain Atlas). To what extent this differentially expressed set of molecules had any role in development of higher cognitive abilities in humans will certainly need further investigation.

Synapse proteome differences at the level of single synapses is also a potential explanation for the observed species differences. The proteomic methods here define composition from large populations of synapses and these populations are known to be made of different types. For example, expression of neurotransmitter receptors (such as NMDA receptor subunits) is distinct on different excitatory synapses. Until synapse proteomic methods can resolve quantitative differences in individual synapses it is unclear if the populations of synapse types in human and mouse neocortex are the same.

The data gathered on the mammalian PSD has prompted us to update and extend our knowledge of the main protein families and signaling pathways likely to be important to PSD function. We have shown that although many protein functions are represented amongst PSD proteins only some are enriched within the PSD suggesting a prominent role for them. These include neurotransmitter receptors, calcium channels, kinases, adaptors, amino acid transporters or small GTPases amongst others. Similarly, we have seen that amongst the many signaling pathways found in the PSD, some are better represented suggesting that they might have a particularly important role. These include the WNT and PI3K pathways, which have been associated with schizophrenia [32], [33], [34]. Finally, PSD molecules show a surprisingly high enrichment in genes involved with Huntington's and Parkinson's disease, although this is not the case for Alzheimer's disease, which in our study seems poorly enriched in PSD proteins although synaptic dysfunction is well characterized as a major symptom of Alzheimer's [43]. In conclusion, these comparative data provide a robust foundation for future comparative studies of mouse and human in the context of evolution and disease.

## Supporting Information

Table S1 Protein Identifications and Proteomic Data. For each identified protein several identification (ID) numbers from biological databases are given. The number of total and uniquely identified peptides for each replicate is also provided. Proteins found with two or more peptides in all replicates are classified as members of the consensus mouse PSD. Human orthologues to mouse proteins are provided. Data regarding human PSD proteins is from: Bayes A et al. Nat Neurosci. 2011 Jan;14(1):19–21.
(XLSX)

Table S2 Human and Mouse PSD Proteins Abundance. Individual and average abundance values (iBAQ) for mouse and human PSD proteins are given. Data was normalized and log2 transformed. Normalization was achieved by dividing abundance data points by its species abundance average. Abundance fold difference was defined as the ratio of mouse to human average abundance values. To measure significant abundance differences between species a Student's t-test was used. Proteins significantly enriched in one species are shown in separate sheets. The abundance of proteins from the postsynaptic protein complexes shown in figure 1 are also shown in a separate sheet.
(XLSX)

Table S3 PSD Proteins Identified Only in One Species. Proteins only found in human or mouse are shown in separate sheets. For each protein several identification (ID) numbers from biological databases are given.
(XLSX)

Table S4 Protein Classes Overrepresented in Proteins Unique to Each Species. Proteins from the total PSD exclusive to human or mouse were classified independently using the Panther ‘Protein Class’ descriptor. Enrichment analysis was done to determine Protein Classes overrepresented in each species set of exclusive molecules. The Benjamini-Hochberg procedure was used to correct for multiple testing. The column ‘observed’ retrieves the number of proteins identified in each ‘Protein Class’ while the column ‘expected’, the number that would have been identified by chance. Over or under-representations are shown by a (+) or (−) symbol respectively. A second sheet contains the same analysis but done only for the molecules from the consensus PSD.
(XLSX)

Table S5 Pathways Overrepresented in Proteins Unique or Enriched to Each Species. Proteins from the consensus PSD exclusive or significantly enriched in human or mouse were classified independently using the Panther ‘Pathway’ descriptor. Enrichment analysis was done to determine Pathways overrepresented in each species set of specific proteins. The Benjamini-Hochberg procedure was used to correct for multiple testing. The column ‘observed’ retrieves the number of proteins identified in each ‘Pathway’ while the column ‘expected’, the number that would have been identified by chance. Over or under-representations are shown by a (+) or (−) symbol respectively.
(XLSX)

Table S6 Biological Processes Overrepresented in Proteins Unique or Enriched to Each Species. Proteins from the consensus PSD exclusive or significantly enriched in human or mouse were classified independently using the Gene Ontology (GO) term ‘Biological Process’. Enrichment analysis was done to determine Biological Processes overrepresented in each species set of specific proteins. The Benjamini-Hochberg procedure was used to correct for multiple testing. The column ‘observed’ retrieves the number of proteins identified in each ‘Biological Process’ while the column ‘expected’, the number that would have been identified by chance. Over or under-representations are shown by a (+) or (−) symbol respectively.
(XLSX)

Table S7 Proteins Related to Axon Guidance or Cell Morphology. Proteins shown are either members of the group of molecules involved in ‘axon guidance mediated by semaphorins’, as defined by Panther, or are known to be involved in this process (literature), or belong to the Biological Process: “cellular component morphogenesis’. Of all molecules belonging to the later group only those that are unique or significantly enriched in human PSD were considered.
(XLSX)

Table S8 Functional Comparison of all PSD Proteins Identified in Human and Mouse. All human and mouse PSD proteins were classified together using the Panther ‘Protein Class’ descriptor. An enrichment analysis was done to determine Protein Classes overrepresented compared with the human genome. The Benjamini-Hochberg procedure was used to correct for multiple testing. The column ‘observed’ retrieves the number of proteins identified in each ‘Protein Class’ while the column ‘expected’, the number that would have been identified by chance. Over or under-representations are shown by a (+) or (−) symbol respectively.
(XLSX)

Table S9 Pathway analysis of all PSD Proteins Identified in Human and Mouse. All human and mouse PSD proteins were classified together using the Panther ‘Pathway’ descriptor. An enrichment analysis was done to determine Pathways overrepresented compared with the human genome. The Benjamini-Hochberg procedure was used to correct for multiple testing. The column ‘observed’ retrieves the number of proteins identified in each ‘Pathway’ while the column ‘expected’, the number that would have been identified by chance. Over or under-representations are shown by a (+) or (−) symbol respectively.
(XLSX)

Table S10 Analysis of dN/dS values from mouse and human PSD proteins. dN, dS, dN/dS and median dN/dS values are given for proteins found in human and mouse cortical PSD, and proteins exclusive or significantly enriched to each species' PSD. All values were obtained from Ensembl 62.
(XLS)

## References

[1] Bayés A, Grant SG (2009) Neuroproteomics: understanding the molecular organization and complexity of the brain. Nat Rev Neurosci 10: 635–646. 19693028 10.1038/nrn2701

[2] Emes RD, Pocklington AJ, Anderson CN, Bayés A, Collins MO, et al (2008) Evolutionary expansion and anatomical specialization of synapse proteome complexity. Nature neuroscience 11: 799–806. 18536710 10.1038/nn.2135 PMC3624047

[3] Husi H, Ward MA, Choudhary JS, Blackstock WP, Grant SG (2000) Proteomic analysis of NMDA receptor-adhesion protein signaling complexes. Nature neuroscience 3: 661–669. 10862698 10.1038/76615

[4] Collins MO, Husi H, Yu L, Brandon JM, Anderson CN, et al (2006) Molecular characterization and comparison of the components and multiprotein complexes in the postsynaptic proteome. J Neurochem 97 Suppl 1: 16–23. 16635246 10.1111/j.1471-4159.2005.03507.x

[5] Dosemeci A, Makusky AJ, Jankowska-Stephens E, Yang X, Slotta DJ, et al (2007) Composition of the synaptic PSD-95 complex. Mol Cell Proteomics 6: 1749–1760. 17623647 10.1074/mcp.M700040-MCP200 PMC2096750

[6] Cheng D, Hoogenraad CC, Rush J, Ramm E, Schlager MA, et al (2006) Relative and absolute quantification of postsynaptic density proteome isolated from rat forebrain and cerebellum. Mol Cell Proteomics 5: 1158–1170. 16507876 10.1074/mcp.D500009-MCP200

[7] Bayés A, van de Lagemaat LN, Collins MO, Croning MD, Whittle IR, et al (2011) Characterization of the proteome, diseases and evolution of the human postsynaptic density. Nature neuroscience 14: 19–21. 21170055 10.1038/nn.2719 PMC3040565

[8] Hahn CG, Banerjee A, Macdonald ML, Cho DS, Kamins J, et al (2009) The post-synaptic density of human postmortem brain tissues: an experimental study paradigm for neuropsychiatric illnesses. PLoS One 4: e5251. 19370153 10.1371/journal.pone.0005251 PMC2666803

[9] Pocklington AJ, Cumiskey M, Armstrong JD, Grant SG (2006) The proteomes of neurotransmitter receptor complexes form modular networks with distributed functionality underlying plasticity and behaviour. Molecular systems biology 2: 2006 0023. 10.1038/msb4100041 PMC1681474 16738568

[10] Gray EG (1959) Electron microscopy of synaptic contacts on dendrite spines of the cerebral cortex. Nature 183: 1592–1593. 13666826 10.1038/1831592a0

[11] Walikonis RS, Jensen ON, Mann M, Provance DW Jr, Mercer JA, et al (2000) Identification of proteins in the postsynaptic density fraction by mass spectrometry. The Journal of neuroscience : the official journal of the Society for Neuroscience 20: 4069–4080. 10818142 10.1523/JNEUROSCI.20-11-04069.2000 PMC6772646

[12] Satoh K, Takeuchi M, Oda Y, Deguchi-Tawarada M, Sakamoto Y, et al (2002) Identification of activity-regulated proteins in the postsynaptic density fraction. Genes to cells : devoted to molecular & cellular mechanisms 7: 187–197. 11895482 10.1046/j.1356-9597.2001.00505.x

[13] Trinidad JC, Thalhammer A, Specht CG, Lynn AJ, Baker PR, et al (2008) Quantitative analysis of synaptic phosphorylation and protein expression. Mol Cell Proteomics 7: 684–696. 18056256 10.1074/mcp.M700170-MCP200

[14] Carlin RK, Grab DJ, Cohen RS, Siekevitz P (1980) Isolation and characterization of postsynaptic densities from various brain regions: enrichment of different types of postsynaptic densities. J Cell Biol 86: 831–845. 7410481 10.1083/jcb.86.3.831 PMC2110694

[15] Cox J, Mann M (2008) MaxQuant enables high peptide identification rates, individualized p.p.b.-range mass accuracies and proteome-wide protein quantification. Nature biotechnology 26: 1367–1372. 10.1038/nbt.1511 19029910

[16] Schwanhausser B, Busse D, Li N, Dittmar G, Schuchhardt J, et al (2011) Global quantification of mammalian gene expression control. Nature 473: 337–342. 21593866 10.1038/nature10098

[17] Flicek P, Amode MR, Barrell D, Beal K, Brent S, et al (2011) Ensembl 2011. Nucleic acids research 39: D800–806. 21045057 10.1093/nar/gkq1064 PMC3013672

[18] Blake JA, Bult CJ, Kadin JA, Richardson JE, Eppig JT (2011) The Mouse Genome Database (MGD): premier model organism resource for mammalian genomics and genetics. Nucleic acids research 39: D842–848. 21051359 10.1093/nar/gkq1008 PMC3013640

[19] Seal RL, Gordon SM, Lush MJ, Wright MW, Bruford EA (2011) genenames.org: the HGNC resources in 2011. Nucleic acids research 39: D514–519. 20929869 10.1093/nar/gkq892 PMC3013772

[20] Hubbard TJ, Aken BL, Ayling S, Ballester B, Beal K, et al (2009) Ensembl 2009. Nucleic Acids Res 37: D690–697. 19033362 10.1093/nar/gkn828 PMC2686571

[21] Thomas PD, Campbell MJ, Kejariwal A, Mi H, Karlak B, et al (2003) PANTHER: a library of protein families and subfamilies indexed by function. Genome research 13: 2129–2141. 12952881 10.1101/gr.772403 PMC403709

[22] Thomas PD, Kejariwal A, Guo N, Mi H, Campbell MJ, et al (2006) Applications for protein sequence-function evolution data: mRNA/protein expression analysis and coding SNP scoring tools. Nucleic acids research 34: W645–650. 16912992 10.1093/nar/gkl229 PMC1538848

[23] Isaac JT, Ashby MC, McBain CJ (2007) The role of the GluR2 subunit in AMPA receptor function and synaptic plasticity. Neuron 54: 859–871. 17582328 10.1016/j.neuron.2007.06.001

[24] Jensen LJ, Kuhn M, Stark M, Chaffron S, Creevey C, et al (2009) STRING 8–a global view on proteins and their functional interactions in 630 organisms. Nucleic acids research 37: D412–416. 18940858 10.1093/nar/gkn760 PMC2686466

[25] Theocharidis A, van Dongen S, Enright AJ, Freeman TC (2009) Network visualization and analysis of gene expression data using BioLayout Express(3D). Nature protocols 4: 1535–1550. 19798086 10.1038/nprot.2009.177

[26] Fernandez E, Collins MO, Uren RT, Kopanitsa MV, Komiyama NH, et al (2009) Targeted tandem affinity purification of PSD-95 recovers core postsynaptic complexes and schizophrenia susceptibility proteins. Mol Syst Biol 5: 269. 19455133 10.1038/msb.2009.27 PMC2694677

[27] Farr CD, Gafken PR, Norbeck AD, Doneanu CE, Stapels MD, et al (2004) Proteomic analysis of native metabotropic glutamate receptor 5 protein complexes reveals novel molecular constituents. Journal of neurochemistry 91: 438–450. 15447677 10.1111/j.1471-4159.2004.02735.x PMC2747775

[28] Hurst LD (2002) The Ka/Ks ratio: diagnosing the form of sequence evolution. Trends Genet 18: 486. 12175810 10.1016/s0168-9525(02)02722-1

[29] Kang HJ, Kawasawa YI, Cheng F, Zhu Y, Xu X, et al (2011) Spatio-temporal transcriptome of the human brain. Nature 478: 483–489. 22031440 10.1038/nature10523 PMC3566780

[30] Yamashita N, Morita A, Uchida Y, Nakamura F, Usui H, et al (2007) Regulation of spine development by semaphorin3A through cyclin-dependent kinase 5 phosphorylation of collapsin response mediator protein 1. The Journal of neuroscience : the official journal of the Society for Neuroscience 27: 12546–12554. 18003833 10.1523/JNEUROSCI.3463-07.2007 PMC6673320

[31] Schmidt EF, Strittmatter SM (2007) The CRMP family of proteins and their role in Sema3A signaling. Advances in experimental medicine and biology 600: 1–11. 17607942 10.1007/978-0-387-70956-7_1 PMC2853248

[32] Koros E, Dorner-Ciossek C (2007) The role of glycogen synthase kinase-3beta in schizophrenia. Drug news & perspectives 20: 437–445. 17992266 10.1358/dnp.2007.20.7.1149632

[33] Brennand KJ, Simone A, Jou J, Gelboin-Burkhart C, Tran N, et al (2011) Modelling schizophrenia using human induced pluripotent stem cells. Nature 473: 221–225. 21490598 10.1038/nature09915 PMC3392969

[34] Okerlund ND, Cheyette BN (2011) Synaptic Wnt signaling-a contributor to major psychiatric disorders? Journal of neurodevelopmental disorders 10.1007/s11689-011-9083-6 PMC3180925 21533542

[35] Li JY, Plomann M, Brundin P (2003) Huntington's disease: a synaptopathy? Trends in molecular medicine 9: 414–420. 14557053 10.1016/j.molmed.2003.08.006

[36] Strand AD, Aragaki AK, Baquet ZC, Hodges A, Cunningham P, et al (2007) Conservation of regional gene expression in mouse and human brain. PLoS genetics 3: e59. 17447843 10.1371/journal.pgen.0030059 PMC1853119

[37] Liao BY, Zhang J (2006) Evolutionary conservation of expression profiles between human and mouse orthologous genes. Molecular biology and evolution 23: 530–540. 16280543 10.1093/molbev/msj054

[38] DeFelipe J, Alonso-Nanclares L, Arellano JI (2002) Microstructure of the neocortex: comparative aspects. J Neurocytol 31: 299–316. 12815249 10.1023/a:1024130211265

[39] Fenstermaker V, Chen Y, Ghosh A, Yuste R (2004) Regulation of dendritic length and branching by semaphorin 3A. Journal of neurobiology 58: 403–412. 14750152 10.1002/neu.10304

[40] Morita A, Yamashita N, Sasaki Y, Uchida Y, Nakajima O, et al (2006) Regulation of dendritic branching and spine maturation by semaphorin3A-Fyn signaling. The Journal of neuroscience : the official journal of the Society for Neuroscience 26: 2971–2980. 16540575 10.1523/JNEUROSCI.5453-05.2006 PMC6673984

[41] Polleux F, Morrow T, Ghosh A (2000) Semaphorin 3A is a chemoattractant for cortical apical dendrites. Nature 404: 567–573. 10766232 10.1038/35007001

[42] Tran TS, Rubio ME, Clem RL, Johnson D, Case L, et al (2009) Secreted semaphorins control spine distribution and morphogenesis in the postnatal CNS. Nature 462: 1065–1069. 20010807 10.1038/nature08628 PMC2842559

[43] DeKosky ST, Scheff SW (1990) Synapse loss in frontal cortex biopsies in Alzheimer's disease: correlation with cognitive severity. Annals of neurology 27: 457–464. 2360787 10.1002/ana.410270502

